# Adequate Neutrophil Responses and Non-Inferior Clinical Outcomes Can Be Achieved by a Two-Day Course of Low-Dose Filgrastim: A Retrospective Single Institution Experience

**DOI:** 10.7759/cureus.1968

**Published:** 2017-12-19

**Authors:** Raj Singh, Henry Heisey, Ahmed G Elsayed, Maria T Tirona

**Affiliations:** 1 Department of Radiation Oncology, Marshall University, Joan C. Edwards School of Medicine; 2 Student, Marshall University, Joan C. Edwards School of Medicine; 3 Hematology and Oncology, Marshall University, Joan C. Edwards School of Medicine; 4 Director of Medical Oncology, Marshall University, Joan C. Edwards School of Medicine

**Keywords:** chemotherapy, neutropenia, g-csf, neutropenic fever, cost savings, quality improvement

## Abstract

Background

Filgrastim is used in the setting of chemotherapy-induced neutropenia to stimulate recovery of bone marrow, which allows for further chemotherapy administration without delay. The recommended dose is 5 ug/kg. The commercially available vials of the drug come in two strengths; 300 ug and 480 ug. Due to these limitations in dosage formulations, it is a frequent occurrence to administer a lower dosage to patients weighing more than 60 kg, in whom the ideal dose would have been more than 300 ug but less than 480 ug. It is also a frequent practice to administer the drug for two consecutive days as it often leads to adequate response that will render patients eligible for their next cycle administration.

Objective

To determine whether a course of 300 ug of filgrastim administered daily for two consecutive days was as successful at reducing chemotherapy-induced neutropenia-related complications in patients with a higher weight (>60 kg) and hence receiving suboptimal dose as compared to those with weight less than 60 kg who are receiving the recommended dose.

Methods

We identified 91 patients from our facility with chemotherapy-induced neutropenia treated with 300 ug of filgrastim daily for two consecutive days, and we separated them into low, medium, and high weight groups. Multivariate logistic regression models examined correlations between outcomes (e.g., increases in absolute neutrophil count) and predictors (e.g., weight groups).

Results

The vast majority of encounters demonstrated rises in white blood cell (WBC) and absolute neutrophil count (ANC). Infection rates were not significantly different between low and medium weight groups (5% vs 0%; p = 0.1658), but the high weight group’s infection rate was significantly higher than the medium weight group (5% vs 33%; p = 0.001). The high weight group did have an increased rate of febrile neutropenia as compared to medium and low weight groups, but these differences were not significant. Incidences of chemotherapy delay and dose reduction were comparable across the three weight groups.

Limitations

Retrospective study, small sample size, heterogeneous cancer sites and different chemotherapy regimens administered limit generalizability of findings.

Conclusion

Patients with weights <85 kg receiving a two-day course of 300 ug of filgrastim have similar neutropenia-related complication rates with a potential percent cost-savings of roughly 43%.

## Introduction

Patients receiving chemotherapy for various malignancies often develop neutropenia and consequent infection resulting in increased morbidity and mortality, compromised treatment outcomes, and excess cost. More than 60,000 cancer patients undergoing chemotherapy are hospitalized for neutropenia each year in the US, with an average cost of $13,372 per hospitalization with associated inpatient mortality [1, 2]. Filgrastim, a pharmaceutical analog of granulocyte colony-stimulating factor (G-CSF), is commonly used to prevent infection in immunocompromised cancer patients by stimulating production of neutrophils by the bone marrow [3].

The therapeutic role of G-CSF analogs in treating febrile neutropenia (FN) is debated. The Infectious Disease Society of America (IDSA) does not recommend G-CSF in the setting of neutropenic fever [4]. However, the role of G-CSF in preventing chemotherapy-induced neutropenia is more established. The American Society of Clinical Oncology (ASCO), the European Society for Medical Oncology (ESMO), the European Organization for Research and Treatment of Cancer (EORTC), and the National Comprehensive Cancer Network (NCCN) suggest consideration for therapeutic use in select patients, particularly those with a 20% or greater risk of developing FN [5-8].

For at least two decades, therapeutic application of G-CSF has been known to reduce the duration of neutropenia even in afebrile patients with severe neutropenia [9]. Colony-stimulating factors like filgrastim have decreased the amount of time patients spend hospitalized, improved their ability to recover from neutropenia, and decreased the duration of antibiotic use, but research has not yet demonstrated a mortality benefit [10]. More recently, secondary intervention with G-CSF for neutropenia has been shown to increase subsequent achievement of adequate chemotherapy dose intensity [11].

Pragmatically, however, the high cost of G-CSF drugs underscores the importance of identifying opportunities for cost savings within the context of the common usage of the drugs. The effective annual per-patient drug cost of filgrastim has been estimated by budget impact models to be between $15,573 and $30,663 [12]. At our own institution, the average wholesale single dose of 300 ug/mL of filgrastim is $1080 as compared to $1889 for a 480 ug vial. As such, patients with weights >60 kg are often times billed for the 480 ug dose despite the fact that they do not require the entire additional dose for the cost incurred. Generally, in our own institution, the same practice is followed, though sometimes patients with weights >60 kg may receive 300 ug of filgrastim rather than the recommended dose that would require a purchase of 480 ug vial. Our facility frequently uses a two-day dosing regimen of G-CSF during subsequent chemotherapy cycles for those patients who had previous delays in their treatment due to neutropenia. As such, we aimed at determining if a two-day course of lower doses of filgrastim demonstrates efficacy for treating established neutropenia and achieves similar clinical outcomes that have previously been reported at the recommended dosage.

## Materials and methods

A retrospective chart review was performed to identify all patients with chemotherapy-induced neutropenia at our institution treated with two consecutive doses of 300 ug of filgrastim between September 13, 2011 and September 13, 2016. Patients were excluded if they did not have a recorded WBC and ANC within two days of the first dose of filgrastim administration. Based on these criteria, a total of 91 patients over 150 encounters were included in the analysis. Relevant patient information was collected and recorded for use as independent variables. These included age (< or > 70 years), weight, race (Caucasian or non-Caucasian), estimated glomerular filtration rate (GFR, < or > 60), primary malignancy site, and the chemotherapeutic agent(s) received.

Regarding weight, patients were divided into three primary groups: low (weight < 60 kg), medium (60 kg < weight < 85 kg), and high (weight greater than or equal to 85 kg). This was done as patients with weights <60 kg receiving 300 ug of filgrastim were receiving the recommended 5 ug/kg dose. Given that the low weight group received the recommended dose, this cohort was treated as the control group for the purposes of this study. As patients in the medium and high weight groups were receiving a sub-therapeutic dose of filgrastim based on label recommendations (as patients with weights >60 kg are recommended to be administered greater than 300 ug of filgrastim), these two cohorts served as the groups of interest. Clinical outcomes relating to chemotherapy-induced neutropenia were examined among the low and medium/high weight groups.

The primary outcomes of our analysis were examining complications relating to neutropenia, including FN, infections, hospitalizations, dose reductions, or missed subsequent chemotherapy cycles during the two weeks following treatment with filgrastim. Hospitalization relating to neutropenia was defined as hospitalization within two weeks of filgrastim treatment secondary to neutropenia, fever, or infection. Primary outcomes were obtained from laboratory findings dated from 1 to 22 days following treatment (mean 6.6 days, standard deviation 3.2 days). Other outcomes were obtained by review of oncologist documentation at our center during the two weeks following filgrastim administration.

Data analysis was performed using Stata 14.0 (StataCorp, College Station, TX). Multivariate logistic regressions were run to determine the effect that potential prognostic factors included in our study had on the probability of relevant outcomes previously discussed.

## Results

A summary of the patient cohort studied can be found in Table 1. The average weight of the cohort was 66.74 kg (range: 41.9–109.6 kg). Thirty of 91 patients had weights less than or equal to 60 kg (low weight group) and thus received the recommended dose of filgrastim. The other 61 patients studied had weights greater than 60 kg and received less than the recommended dose, with 53 patients weighing between 60 and 85 kg (medium weight group) and eight patients with weights greater than 85 kg (high weight group). Twenty-four patients (26%) were receiving chemotherapy for breast cancer, 16 for colorectal cancer (18%), 33 for lung cancer (36%), and the remaining 18 (20%) for cancers of other sites. The mean total dose in ug/kg that was administered over the course of two days was 7.37 (SD: 2.28) for patients with weights >85 kg, 8.38 (SD: 3.11) for those in the medium weight group, and 11.19 (SD: 4.58) for patients with weights <60 kg.

**Table 1:** Patient characteristics. BMI: Body mass index; GFR: Glomerular filtration rate.

Variable	Summary Statistics
Total Patients	91 patients
Patients with weight ≥ 85 kg	8 patients
Patients with 60 kg < weight < 85 kg	53 patients
Patients with weight ≤ 60 kg	30 patients
Total Filgrastim Administrations	150 encounters
Encounters with weight ≥ 85 kg	12 encounters
Encounters with 60 kg < weight < 85 kg	91 encounters
Encounters with weight ≤ 60 kg	47 encounters
Average Dose by Weight Group (ug/kg)	
Patients with weight ≥ 85 kg	7.37 (SD: 2.28)
Patients with 60 kg < weight < 85 kg	8.38 (SD: 3.11)
Patients with weight ≤ 60 kg	11.19 (SD: 4.58)
Gender	
Male	23
Female	68
Age (range)	60.64 (33 – 87)
Race	
Caucasian	73 patients
Hispanic	1 patient
Asian-American	1 patient
African-American	3 patients
Unknown	13 patients
Height (cm) (range)	165.79 (149 – 185.4)
Weight (kg) (range)	66.74 (41.9 – 109.6)
BMI (range)	24.31 (16.03 – 41.8)
GFR (range)	74.49 (26.59 – 135.69)
Malignancy Site	
Breast	24 patients
Colorectal	16 patients
Lung	33 patients
Other	18 patients
Chemotherapy Received	
Paclitaxel	24 patients
Gemcitabine	11 patients
Cisplatin	16 patients
Carboplatin	22 patients
Folfox	21 patients
Doxorubicin	6 patients
Bevacizumab	14 patients
Etoposide	12 patients

Outcomes for the entire patient cohort studied can be found in Table 2. The average elapsed time between filgrastim administration and repeat complete blood count (CBC) was 6.63 days (range: 1 day to 22 days). Following administration of filgrastim, 98% and 95.33% of encounters had documented rises in WBC and ANC, respectively. Patients in the low, medium, and high weight groups had similar WBC responses to filgrastim, as 95.74%, 98.9%, and 100% of such encounters had documented rises in WBC, respectively. Similar results were noted for the low (95.74%), medium (94.51%), and high (100%) weight groups with regards to ANC response rates.

**Table 2:** Summary of both laboratory and clinical responses over 150 patient encounters following administration of a shortened course of low-dose filgrastim. ANC: Absolute neutrophil count; CBC: Complete blood count; FN: Febrile neutropenia; WBC: White blood cell.

Variable	Summary Statistics
Average number of days between filgrastim administration and repeat CBC (range)	6.63 (1 to 22)
Encounters with rise in WBC	Weight < 60 kg - 95.74% 60 kg < Weight < 85 kg - 98.9% Weight > 85 kg - 100%
Encounters with rise in ANC	Weight < 60 kg - 95.74% 60 kg < Weight < 85 kg - 94.51% Weight > 85 kg - 100%
Complication Rates	
Instances of infection within two weeks of encounter	Weight < 60 kg – 0% 60 kg < Weight < 85 kg - 5.49% Weight > 85 kg - 33.33%
Instances of FN-related hospitalization within two weeks of encounter	Weight < 60 kg – 0% 60 kg < Weight < 85 kg – 3.33% Weight > 85 kg – 8.33%
Instances of patients having to miss next cycle of chemotherapy	Weight < 60 kg - 25.53% 60 kg < Weight < 85 kg - 24.18% Weight > 85 kg - 25%
Instances of patients having dose reduction	Weight < 60 kg - 6.38% 60 kg < Weight < 85 kg - 5.49% Weight > 85 kg - 8.33%

A summary of incidences of FN-related hospitalizations, incidences of infections, dose reductions, and treatment delays can also be found in Table 2. Patients in the high weight group were noted to have a markedly higher rate of infection two weeks following filgrastim administration (33%) as compared to the low weight (0%) and medium weight (5.49%) groups. The association between having a high weight and an increased likelihood of infection was statistically significant (p = 0.001; Table 3). A similar trend was found with regards to incidences of FN-related hospitalizations, which were highest among the higher weight group (8.33%) as compared to the medium (3.33%) and low weight groups (0%). However, this was not found to be significant (p = 0.239). None of the prognostic factors included in our analysis were noted to be correlated with requiring hospitalization secondary to FN. Roughly 6%, 5.5%, and 8% of patients in the low, medium, and high weight groups, respectively, required dose reductions due to neutropenia after receiving filgrastim. Weight was not correlated with incidences of dose reductions. Nearly 25% of patients in each weight group also experienced delays in treatment and none of the variables analyzed were significantly associated with treatment delays.

**Table 3:** Multivariate logistic regressions examining impact of potential prognostic factors on neutropenia-related complications following filgrastim administration. * = p < 0.05, ** = p < 0.01. FN: Febrile neutropenia; GFR: Glomerular filtration rate.

Variable	p-value (Febrile neutropenia-related hospitalization)	p-value (Infection)	p-value (Treatment Delay)	p-value (Dose Reduction)
60 kg < Weight < 85 kg	0.559	0.1658	0.863	0.747
Weight ≥ 85 kg	0.239	0.001**	0.978	0.724
Gender	0.185	0.372	0.609	0.372
Age > 70	0.962	0.898	0.620	0.503
Race (Non-Caucasian)	0.987	0.169	0.412	0.893
GFR < 60	0.962	0.034*	0.697	0.152

WBC and ANC responses for the cohort broken down by weight can be found in Table 4. A similar number of patients in the low, medium, and high weight groups had documented rises in WBC counts (95.74%, 98.9%, and 100%) and ANC counts (95.74%, 94.51%, and 100%, respectively) with all being significant increases (p < 0.0001) following paired t-tests.

**Table 4:** Outcomes in different weight groups. ANC: Absolute neutrophil count; WBC: White blood cells.

Variable	Weight ≤ 60 kg	60 kg < Weight < 85 kg	Weight ≥ 85 kg
WBC Counts			
Average Pre-Filgrastim WBC (range)	2270 (500 to 4500)	1832 (500 to 5200)	2258 (1000 to 4400)
Average Post-Filgrastim WBC (range)	7302 (600 to 23,600)	7281 (900 to 36,300)	6508 (2700 to 12,500)
Average Rise in WBC (range)	5032 (-600 to 21,000)	5389 (-1300 to 34,200)	4250 (100 to 10,600)
Average Percent Change in WBC (range)	352% (77% to 14,000%)	494% (41% to 18,686%)	348% (103% to 6800%)
Encounters with rise in WBC	95.74%	98.9%	100%
ANC Counts			
Average Pre-Filgrastim ANC (range)	675 (100 to 1500)	638 (84 to 1600)	950 (200 to 1900)
Average Post-Filgrastim ANC (range)	4801 (300 to 20,500)	5173 (307 to 33,000)	4943 (1400 to 10,100)
Average Rise in ANC (range)	4127 (-600 to 19,900)	4534 (-1290 to 32,000)	3993 (200 to 9500)
Average Percent Change in ANC (range)	1011% (33% to 53,500%)	1365% (19% to 62,000%)	728% (117% to 24,500%)

## Discussion

There have been limited reports examining whether 300 ug of filgrastim is as efficacious as the recommended dose of 5 ug/kg, particularly for patients with weights above 60 kg. As such, this retrospective single-institution study aimed at examining whether patients receiving 300 ug of filgrastim experience similar incidences of FN, neutropenia-related infections, and dose reductions or treatment delays as previous reports in the literature that prescribed patients a 5 ug/kg dose. Based on our findings, patients in the medium weight cohort (that received a sub-therapeutic dose) had similar WBC/ANC responses as well as neutropenia-related outcomes as compared to those in the low-weight cohort (that received the recommended dose). However, given the significantly higher infection rate among the high-weight group, patients with weights >85 kg seem to have inferior outcomes with the administration of low-dose filgrastim.

There have been many other studies in the literature examining the impact of filgrastim and similar short-acting G-CSF analogs in reducing incidences of FN as well as hospitalizations relating to FN following administration at the recommended dose of 5 ug/kg. A multicenter retrospective review with a cohort of patients with a wide range of cancer sites (i.e., lung, GI, GU, head and neck, etc.) treated with filgrastim for a median range of five days has previously reported 10.9% of patients experiencing FN-related hospitalizations (23/211) [13]. In one of the largest multicenter studies representing a variety of cancer sites conducted to date, patients receiving a mean of 5.2 and 3.7 days of consecutive filgrastim treatments in 2001 and 2003, respectively, were reported as having FN incidences of 5.3% (31/583) and 7.3% (63/868) [14]. Other retrospective studies as well as randomized trials examining the use of filgrastim and other short-acting G-CSFs for breast cancer patients receiving adjuvant or neoadjuvant chemotherapy reported incidences of FN in their cohorts ranging from 9.1% to 18% [15-17]. One study with similar findings to our own found FN incidence among Stage II to IV breast cancer patients treated with filgrastim (the majority receiving at least nine injections) to be 2.38% [18]. As such, the shorter course of low dose filgrastim administered among the medium weight cohort at least achieved similar rates of FN-related hospitalization (3.3%) reports that have been published for patients receiving the recommended dose.

With regards to dose reductions and delay, approximately 6%, 5.5%, and 8% of patients in the low, medium, and high weight groups required dose reductions and roughly 25% of patients among each of the weight groups had treatment delays. These findings also seem to be consistent with the literature. In a cohort study of 239 breast cancer patients that were administered filgrastim over a median of seven days (range: 4-10 days), 17% of patients required a delay of treatment and 19% required a dose reduction [19]. Another study examining the possible benefit of filgrastim among 1058 breast cancer patients reported that 18.5% and 42% of patients receiving filgrastim required a dose reduction or treatment delay, respectively [20]. In comparison to prior reports, 300 ug of filgrastim for patients in our study with weights <85 kg seemed to produce a non-inferior outcome.

As another area of interest, we analyzed the cost-savings of administering the two-day course of low dose of filgrastim as compared to a two-day course at the recommended dose that would necessitate the purchase of a 480 ug dose. Given that the medium weight group had similar clinical outcomes to both the low weight group in our study as well as other cohorts reported in the literature, we limited our analysis to this subset. Over 91 encounters for the medium weight cohort and assuming a wholesale cost of $1889.20 for a 480 ug dose as compared to $1080 for a 300 ug dose (representing a nearly 43% cost savings per dose), the total cost savings for this cohort alone translates to $147,274.40. Given the small size of this cohort, these cost savings would no doubt have significant implications at a national level if non-inferiority is able to be further corroborated. Also, it is important to note that this analysis only takes into account potential savings from the lower dosage utilized but not from a two-day versus the typical multiple-day dosing.

There are limitations to our study that merit attention. First, the total patient cohort was small, especially when dividing among different weight groups, which limits both the power and generalizability of our findings. Second, there is the possibility of under-reporting, particularly with regards to incidences of FN and subsequent hospitalizations. Our institution serves as a tertiary care provider for a large geographic area, and as such patients seen in our outpatient clinics may have been admitted to other hospitals for FN that we were unable to account for in our analysis. Similarly, even if patients reported having been hospitalized for FN or infection following chemotherapy at outside institutions, we did not report on the severity and duration of FN-related hospitalizations. Also, the variety of cancer sites that the patients represented as well as varying chemotherapy regimens administered also limits the generalizability of our findings. Another limitation is the lack of control for other comorbid conditions that might have impacted the likelihood of infection or FN risk. It is also not ideal to have unequal time intervals between the repeat CBC and filgrastim administration, though roughly 68% of patients had a repeat CBC within roughly one standard deviation (roughly 3-10 days based on a mean of 6.6 days and a standard deviation of 3.2 days). Even though some patients did have repeat CBC outside of this time frame, we believe other reported outcomes such as infections and hospitalization are representative of immune response as well. Finally, the retrospective nature of our study and lack of inclusion and randomization between the lower dose of 300 ug and the recommended 5 ug/kg among similar weight groups make comparison of outcomes between these two regimens difficult.

## Conclusions

The results of this retrospective single institution study have demonstrated non-inferior outcomes with regards to incidences of FN-related hospitalizations, infections, dose reductions, and treatment delays utilizing a two-day course of 300 ug of filgrastim for patients with weight <85 kg. Based on this observation, dosing filgrastim at 300 ug may be appropriate in this weight group with a roughly 43% cost reduction in our experience. Prospective randomized trials are warranted to validate these findings in a larger uniform cohort controlled for both disease site as well as chemotherapy received.
